# Auxiliary signal-guided knowledge encoder-decoder for medical report generation

**DOI:** 10.1007/s11280-022-01013-6

**Published:** 2022-08-27

**Authors:** Mingjie Li, Rui Liu, Fuyu Wang, Xiaojun Chang, Xiaodan Liang

**Affiliations:** 1 University of Technology Sydney, Sydney, Australia; 2 Monash University, Melbourne, Australia; 3 Sun Yat-sen University, Guangzhou, China; 4 Sun Yat-sen University, Shenzhen, China

**Keywords:** Medical report generation, Auxiliary signals, Transformer, Generative pre-training

## Abstract

Medical reports have significant clinical value to radiologists and specialists, especially during a pandemic like COVID. However, beyond the common difficulties faced in the natural image captioning, medical report generation specifically requires the model to describe a medical image with a fine-grained and semantic-coherence paragraph that should satisfy both medical commonsense and logic. Previous works generally extract the global image features and attempt to generate a paragraph that is similar to referenced reports; however, this approach has two limitations. Firstly, the regions of primary interest to radiologists are usually located in a small area of the global image, meaning that the remainder parts of the image could be considered as irrelevant noise in the training procedure. Secondly, there are many similar sentences used in each medical report to describe the normal regions of the image, which causes serious data bias. This deviation is likely to teach models to generate these inessential sentences on a regular basis. To address these problems, we propose an Auxiliary Signal-Guided Knowledge Encoder-Decoder (ASGK) to mimic radiologists’ working patterns. Specifically, the auxiliary patches are explored to expand the widely used visual patch features before fed to the Transformer encoder, while the external linguistic signals help the decoder better master prior knowledge during the pre-training process. Our approach performs well on common benchmarks, including CX-CHR, IU X-Ray, and COVID-19 CT Report dataset (COV-CTR), demonstrating combining auxiliary signals with transformer architecture can bring a significant improvement in terms of medical report generation. The experimental results confirm that auxiliary signals driven Transformer-based models are with solid capabilities to outperform previous approaches on both medical terminology classification and paragraph generation metrics.

## Introduction

When you take a medical image in any hospital, you will receive a medical report. This medical report describes both normal and abnormal terminologies, and can assist radiologists and specialists in diagnosing and reviewing. However, writing medical reports is error-prone and time-consuming, especially during a pandemic like COVID-19, because radiologists may have to diagnose hundreds of images per day. Therefore, the topic of automatically generating medical reports has attracted research attention from both artificial intelligence and clinical medicine fields.

The most similar task to medical report generation in the computer vision field is image captioning. Beyond the common difficulties in natural image captioning, there are three more bottlenecks for medical report generation. Firstly, the amount of image-report pairs in existing datasets are considered small compared to the captioning datasets, which are insufficient to learn visual representations; Secondly, it is hard to acquire the object features which are widely used in the natural image captioning tasks [1] from medical images. Only a few medical images can provide the well-annotated segmentation or location information of lesions; Thirdly, there are severe data deviation exists in these datasets. Some diseases are rare in nature, and their positive samples are hard to collect. Moreover, there are many similar sentences used in each report to describe the routine observation, which leads to the overfitting problem and limits the generalization of neural approaches [18, 21, 33, 34].

Recently, many approaches have been designed to address these problems and achieved promising performance on automatically generating medical reports [3, 12, 17, 21]. For example, Xue et al. [40] encode multiple image modalities to generate multi sentences. Li et al. [21] manually proposed several templates and Zhang et al. [45] encode and modeled visual contents relationships by the incorporation of graph module to generate fine-grained reports. With the success of Transformer [36] in image captioning tasks, Chen et al. [3] firstly proposed a memory-driven Transformer that can update the memory during generating process. Although achieving promising performances, R2Gen [3] focuses on designing extra modules, ignoring activating the characteristic learning ability of Transformer. Although achieving promising performances, existing approaches did not fully activate neural models’ potentiality, especially Transformer.

**Fig. 1:**
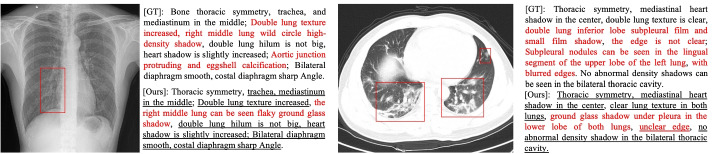
Two samples from CX-CHR and our COV-CTR datasets. Red bounding boxes annotated by a radiologist indicate the regions that he pays more attention to describing this image. The red text describes the abnormalities. Underlined text indicates alignment between ground truth reports and generated reports

Inspired by the radiologists’ working patterns, in this paper, we explore auxiliary signals’ power to facilitate generating medical reports. Generally, when a radiologist describes a medical image, he/she will carefully inspect the suspicious regions after quickly browsing the global image. Then, he/she will write a report that draws on the knowledge he/she learned from the external medical domain and his/her working experience. As shown in Fig. 1, the suspicious region takes up only a tiny portion of the global image but has been treated equally to other regions in previous works. Therefore, other regions could be considered irrelevant noise that distracts the model. Although these regions may get more attention based on the self-attention mechanism in Transformer, Dosovitskiy et al. [6] pointed out that Transformer can learn a better visual representation when fed with original image patches instead of the encoded visual features. Using large extra corpora to pre-train the Transformer is an effective way to alleviate the corpus deviation in the training datasets [5, 31]. However, there is a considerable textual semantic gap between the medical and common domains.

Accordingly, to mimic the behavior of medical experts and address the above-mentioned learning difficulties, we propose an Auxiliary Signal-Guided Knowledge (ASGK) approach including two kinds of auxiliary signals to improve a Transformer to generate medical reports. Firstly, we automatically find a suspicious region where the pre-trained neural visual extractor paid the most attention. After resizing and cutting, the auxiliary patches are concatenated to the original patch features before being fed to the encoder. These patches ensure that the Transformer will learn better visual hidden representations. Then, we collect a medical corpus to pre-train the decoder, in which all the sentences that record related medical knowledge are easily accessed online. The pre-training steps can improve the model robustness to alleviate the training corpus deviation and decrease the sensitivity to similar linguistic patterns.

We further introduce a new COVID-19 CT Report (COV-CTR) dataset for use in validating the robustness and generalization ability of ASGK. Since December 2019, the novel COVID-19 virus has caused a global pandemic and infected millions of people across 200 countries. A key step in controlling the infection is that of identifying infected people. In addition to the Reverse Transcription Polymerase Chain Reaction (RT-PCR) tests, lung CT scan analysis has emerged as another essential testing method. Therefore, an accurately written report could assist patients and doctors to understand their health condition. We invited three radiologists with more than five years of working experience to apply their diagnostic skills to the public COVID-CT dataset [47] and use this information to construct the COV-CTR dataset.

We test our approach on the large-scale Open-IU [4], CX-CHR dataset [21] and our COV-CTR dataset. We adopt CIDER-D [37], ROUGE-L [23] and BELU [28] as the metrics for evaluating our approach. Comprehensive experiments demonstrate that ASGK improves performance in terms of both tag classification and report generation. Our ablation studies also provide insight that enables us to determine how ASGK works well.

The main contributions of this paper are three-fold as follows:

- We identify and produce two kinds of auxiliary signals, namely the internal fusion visual features and the external medical linguistic information to facilitate graph encoding and medical knowledge learning respectively.
- We design a medical tag graph encoder to transfer input features into higher-level information and adopt Generative Pre-Training (GPT) [31] as our natural language decoder to generate accurate and robust medical reports.
- We invite three radiologists with more than five years of experience to apply their diagnostic skills to the COVID-19 CT images [47] and use this information to construct a new medical report dataset, COVID-19 CT Report which will be available.

## Related work

### Medical report generation

Compared with natural image captioning [1, 22], medical reports generation is a more challenging research topic. The models should have the capability to detect all visual groundings, classify them accurately, and generate multi-sentences to describe both normal and abnormal terminologies. Most existing medical report generation approaches are based on the encoder-decoder frameworks. At the beginning, Jing et al. [13] firstly proposed a data-driven neural network composing of a convoluational neural network and a LSTM [10] to simultaneously predict medical tags and generate a single sentence report by employing a co-attention mechanism over both visual and textual features. To generate multi-sentences, Xue et al. [40] proposed a multi-level recurrent generation model that consists of a topic level LSTM and a word-level LSTM, they also concentrated the front and later views to fuse multiple images modalities. These concepts have been regarded as successful practices and employed by the following works [7, 21, 45]. Compared with the LSTM, Transformer [36] has great effectiveness in processing long sequence information. Thus, Chen et al. [3] proposed a memory-driven Transformer to generate the report, in which the critical information from the previous generation process can be incorporated into the Transformer. In contrast, Wang et al. [39] proposed a region-level extractor instead of the global features by a selective search algorithm.

Beyond designing backbone networks, prior medical knowledge is another resource to advance the medical report generation researches. Most recently, Liu et al. [24] also explored radiologists’ working patterns which is similar to our work. In particular, they enhanced the report generation procedure by retrieving the similar reports according to the input visual features. While we utilized the external medical knowledge to alleviate the textual bias, more evidence of our advantages are provided in the experimental section.

### Medical image analysis with auxiliary signals

With success if deep learning networks in many fields [16, 20, 46], recent works [11, 35] discussed the application of deep learning technologies to the field of medical image analysis. Medical knowledge graph is adopted as a kind of prior knowledge to facilitate the medical image analysis and achieves significant improvements [2, 9, 17, 24, 26, 27, 42, 44, 45, 48]. However, due to the difficulty associated with accessing and annotating medical images, many researchers have attempted to use self-supervised learning to loosen the requirements of training data. The core of self-supervised learning involves the design of various proxy tasks that provide auxiliary signals for training deep neural networks [14]. Furthermore, auxiliary signals are widely applied as the basic structure for image analysis. Adopting auxiliary signals to guide training has advantages in terms of boosting model performance and improving model robustness. Zhuang et al. [49] found that auxiliary signals are likely to benefit 3D neural networks for brain hemorrhage classification and brain tumor segmentation.

### Language model pre-training

Natural language decoders are another critical part of the image captioning process. Recent breakthroughs in the field of pretrained language models, such as ELMO [30], BERT [5], and XLNet [41], have demonstrated the effectiveness of auxiliary signals for a widespread range of natural language processing tasks. For example, the new state-of-the-art GPT-2 [32] reveals that pretraining allows models to learn a language’s syntactic and semantic information via unsupervised learning, which is then transferred to other tasks. However, directly applying these models to medical domain datasets often yields unsatisfactory results due to the existence of a domain gap between general corpora and medical corpora. To tackle this problem, Habibi et al. [8] proposes a completely generic method based on deep learning and statistical word embedding, while Lee et al. [15] pretrains BERT on medical corpora.

## Approach

### Problem setup

Similar to the previous studies [13, 17, 21, 45], the task of medical report generation involves asking a model to generate a topic related paragraph consisting of a series of sentences to describe a medical image of a patient case. We represent the image as *I* and the report as $S=\left\{ w_1, w_2,..., w_l|w_i\in \mathbf{V} \right\}$, where $w_i$ presents the index of word in $\mathbf{V}$ the vocabulary of all words contained in the datasets. To generate fine-grained and semantically coherent sentences, we propose a graph encoder-decoder framework that first encodes inputs feature vectors to a medical tag graph and then decodes them to a medical report. We represent the medical tag as $G=(V, E)$, where $V=\left\{ v_i\right\} _{i=1:N_t}$ and $E=\left\{ e_{i,j}\right\} _{i,j=1:N_t}$ is a set of edges. In our task, we represent each node feature $v_i$ by its detected tag classification probability, then encode the correlation between each of the two tags as edge weights. $N_t$ represents the total number of medical tags composes abnormal terminologies, such as “pneumothorax” and “colon shadow”, and normal terminologies such as “normal spine”, “normal intercostal space” and so on.

Generally, when a radiologist describes a image, he will inspect the abnormal region carefully after quickly browsing the global image, then write a report that reflects both his inspection and the knowledge obtained from external medical domain information and his working experience. To mimic this pattern, we firstly pretrain the framework with the external medical signals collected from an appropriate website in order to correctly phrase and learn medical knowledge. Subsequently, the internal visual fusion signals facilitate graph encoding and bridge the gap between linguistic and visual domain. More details regarding these internal visual fusion signals are described in Section 3.3.

### The structure of ASGK

**Fig. 2:**
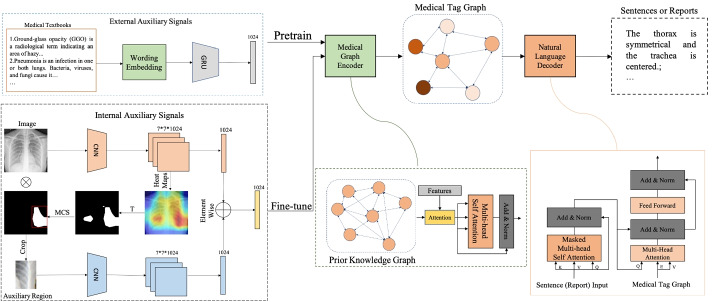
An overview of our ASGK approach. The ASGK model consists of a medical graph encoder and a natural language decoder. The medical graph encoder encodes input features into the corresponding medical tag graph, while the natural language decoder transfers high-level information to sentences or reports. The external signals guide the pretraining procedure, while the internal signals guide the model to bridge linguistic and visual information. T and MCS represent threshold and max connection select operation respectively

An overview of our approach is shown in Fig. 2. The main structure of ASGK comprises a medical graph encoder and natural language decoder.

#### Medical graph encoder

This component is built to encode the input features into higher level information, i.e. a medical tag graph. In the medical graph, each node denotes one detected medical tag, the features of which are the classification probabilities and can be written as (1).

1 $$\begin{aligned} V = \mathrm{Sigmoid}(\mathrm{W}_\mathrm{v}\mathrm{f}_\mathrm{input}) \end{aligned}$$

where $W_{v}$ is a projection matrix of size $N\times {d}$; here, *d* represent the dimension of the input features, and N is the number of total tags. Given that the truth edge information is not available in our case, we conduct an attention operation to learn edge weights automatically, which can be written as follows:

2 $$\begin{aligned} e_{i,j} = \mathrm {Norm}(\mathrm {Attention}(W_{v}v_i, W_{v}v_j)) \end{aligned}$$

where *Norm* is the normalization operation, while *Attention* is executed as a scaled dot-product operation. Then the medical tag graph is incorporated with the prior medical knowledge which is represented as a set of nodes of size N with initialized features and edges via attention mechanism following by [17], which can be written as follows:

3 $$\begin{aligned} G = \mathrm {att}(G_{prior}, V, E) \end{aligned}$$

To enhance the correlation between each of the nodes, we employ a multi-head self attention operation on *G* to get the final graph. We further treat medical tag detection as a multi-label classification task and adopt BCE loss to maximize the prediction scores

4 $$\begin{aligned} L_{tagcls} = -\sum \limits ^{N-1}_{i=0}y_i\log {v_i}+(1-y_i)\log (1-v_i) \end{aligned}$$

where $W_{v}$ is a projection matrix of size $N\times {d}$; here, *d* represent the dimension of the input features, $y_i$ is the ground truth label, and $v_i$ is the final graph tag features.

#### Natural language decoder

Inspired by GPT [31], we design a natural language decoder consisting of $N=3$ blocks, similar to the Transformer decoder, to interpret the medical tag graph and enable semantic alignment in the visual and linguistic domain. The structure of the block is presented in Fig. 2. This block applies a masked, multi-head self-attention operation to the medical report or sentences tokens $T=\left\{ t_1, t_2,..., t_l\right\}$ embedded from Glove vectors pretrained on our datasets. We use [31] to maximize the likelihood in the following formulation:

5 $$\begin{aligned} L_t(T) = -\sum \limits _{i}\log {P(t_i|t_1,...,t_{i-1}; \varTheta )} \end{aligned}$$

where *P* is the conditional probability of the next token prediction, modeled using a neural network with parameters $\varTheta$ and history sentences. Then, followed by position-wise feed forward layers, the natural language decoder aims to produce an output distribution over all token vocabulary.

6 $$\begin{aligned} h_0= & {} I_WW_e+I_PW_p, \end{aligned}$$

7 $$\begin{aligned} H_l= & {} \mathbf{block} (h_{l-1}, V, E) \forall {l}\in [1, N], \end{aligned}$$

8 $$\begin{aligned} P_i= & {} \mathrm {Softmax}(h_NW^T_e) \end{aligned}$$

where $I_W$ is the index of input tokens in the vocabulary, $I_P$ is the index of the token’s position, $W_e$ is the pretrained wording embedding matrix, and $W_p$ is the position embedding matrix.

### Auxiliary signal-guide learning

#### Pretraining with external auxiliary signals

The direct application of general pretrained language models to medical domain tasks leads to unsatisfactory results, since the word distributions differ from those of those of general and medical corpora. To resolve this problem, we collect medical textual information from an appropriate website to construct a large-scale medical textbook. This textbook provides sufficient information about medical knowledge, including the symptoms, manifestations and other information about COVID-19 and thoracic diseases. Before feeding it into the medical graph encoder, we divide the medical textbook into sentences and embed the word tokens with embedding vectors, which are trained in our datasets using Glove [29]. After embedding, sentences are encoded using a single-layer GRU with 1024 hidden units to produce the external medical auxiliary signals.

#### Training with internal auxiliary signals

Evidently, the quality of the encoded medical graph will significantly affect the accuracy of the generated reports. Therefore, we produce internal fusion visual signals to facilitate medical graph encoding and bridge the gap between linguistic and visual information. As shown in Fig. 2, we first classify the global image using DenseNet-121 and obtain the feature maps $f_{c}\in {R^{7*7*1024}}$ before the final pooling layers and output from last pooling layers $f_{g}\in {R^{1*1024}}$. To produce the mask, we perform a threshold operation on a heat map acquired by (9) and select the max connected area:

9 $$\begin{aligned} H = \underset{k}{\max }\,\left( |f_{c}^{k}|\right) , k\in {1:0124} \end{aligned}$$

We adopt another DenseNet to extract the attended region features $f_{l}\in {R^{1*1024}}$ from the final pooling layers, then perform the element-wise operation on $f_g$ and $f_l$ to produce the fusion signals $f_f$. To balance the deviation in medical tags, we optimize the parameters of three branch via focal loss, as follows:

10 $$\begin{aligned} p^*_i= & {} \left\{ \begin{array}{cc} p_i,&{}\;\mathrm{if\;\;\;y_i=1} \\ 1-p_i,&{}\;\mathrm{otherwise} \end{array}\right. \end{aligned}$$

11 $$\begin{aligned} L_{focal}= & {} -\sum \limits ^{N-1}_{i=0}\alpha (1-p^*_i)^\gamma \log {p^*_i} \end{aligned}$$

where $y_i$ represents the label, $p_i$ represents the prediction probability, $\alpha$ is a hyper-parameter set according to diverse datasets, and $(1-p^*_i)^\gamma$ is treated as a modulating factor with a tunable focusing parameter $\gamma \ge 0$. We set $\alpha$ to 0.25 and $\gamma$ to 2 in our task.

## Experiments

### Datasets

We invited three Chinese radiologists with more than five years of working experience to apply their diagnostic skills to the public COVID-CT [47] and use these image-report pairs to construct the COV-CTR. All the images are lung CT-scans and collected from the published papers. The references to these papers are listed in [47]. Notably, the quality of these images are degraded in following aspects: the Hounsfield unit (HU) values are lost; the number of bits per pixel is reduced; the resolution of images is reduced. However, as explained in [47], experienced radiologists are able to make an accurate diagnosis from low quality CT images. For example, given a photo taken by smart phone of the original CT image, experienced radiologists can make an accurate diagnosis by just looking at the photo, though the CT image in the photo has much lower quality than the original CT image. Likewise, the quality gap between CT images in papers and original CT images will not largely hurt the accuracy of diagnosis.

For each image in COV-CTR, we present the related reports and the impression which indicates the patient is COVID or not. There are 349 and 379 images for COVID and Non-COVID, respectively. More details and comparisons with other datasets are reported in Table 1 Medical report generation tasks aim to describe all the visual grounding in the image with medical terminologies. Therefore, one CT scan is enough for neural models to diagnose.

**Table 1:** Statistics of COV-CTR, CX-CHR and Open-IU

Statistics	COV-CTR	CX-CHR	IU X-Ray
Patients	−	35,609	3867
Images	728	45,598	7470
Normalities	−	18	−
Abnormalities	−	155	−
Vocabulary Size	235	27683	2791
Max. Sen. Num.	14	24	18
Max. Sen. Len.	37	38	42
Max. Rep. Len.	127	216	173
Avg. Sen. Len.	8.197	7.111	6.997
Avg. Rep. Len.	77.274	64.858	32.450

We conduct experiments on both Chinese annotated CX-CHR, COV-CTR dataset and English described Open-IU dataset in order to validate the robustness and generalization ability of ASKG. CX-CHR is a large-scale chest X-ray dataset, constructed by a professional medical institution, that consists of 35,609 patients and 45,598 images paired with their corresponding Chinese diagnostic reports. We collect 173 medical tags comprising 155 abnormal terminologies and 28 normal terminologies from the ‘findings’ section and annotate paired images with these tags. Moreover, the COV-CTR datasets consist of 728 images (349 for COVID-19 and 379 for Non-COVID) collected from published papers and their corresponding paired Chinese reports. We perform the same operation described above and collect 68 tags (50 abnormalities and 18 normalities). We adopt the same Chinese textbook when conducting experiments on two Chinese datasets. We tokenize all reports and the medical textbook and filter tokens with a minimum frequency of three, which results in 27683 unique Chinese tokens covering over $98.7\%$ of words in the corpus including four special tokens *pad*, *eos*, *sep* and *unk*. On both Chinese datasets, we randomly split the data into training, validation, and testing sets using a ratio of 7 : 1 : 2; there is no overlap between these branches.

We perform the same operations on the Open-IU dataset to clarify the performance of our ASKG to generate English medical reports, we collected medical papers’ abstracts from Pubmed to construct the English Medical Textbook and provide the external signals with 2791 unique English tokens. Then we included 20 finding keywords as disease categories the same as [45] to extract the internal signals.

### Evaluation metrics

**Fig. 3:**
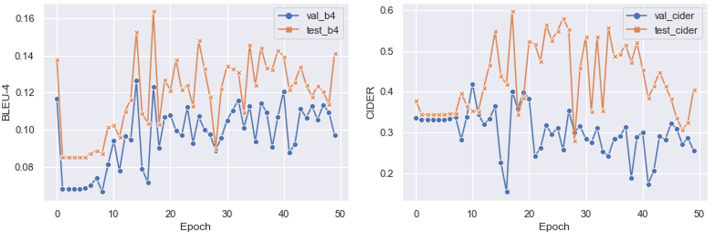
We evaluate our model each epoch and report BLEU-4 and CIDER values on validation and testing sets

Following [17], we adopt three kinds of metrics to evaluate our approach. Firstly, we use area under the curve (AUC) to evaluate the performance of all medical tag classifications. We compare our approach with existing approaches, including conventional natural image captioning models and typical medical report generation pipelines on the metrics including CIDER-D [37], ROUGE-L [23], BLEU [28] and clinical efficacy. Most existing medical report generation approaches adopt the BLEU-4 as the primary metric. However, as shown in Fig. 3, the model achieves a high BLEU value in the first epoch, where all outputs of models are the same. Obviously, BLEU has limits on evaluating medical reports. Compared with BLEU, CIDER pays more attention to the different words between each sentence, and most of the words describe abnormal terminologies in this task. Therefore, we adopt the CIDER as our primary metric. As discussed in [19, 45], these metrics can not provide reliable evaluation results. We also conduct human evaluation, inviting senior radiologists to judge the quality of generated reports. Specifically, we randomly select 200 samples from the testing set and generate corresponding medical reports using CoAtt [13] and our approach. Then we invite senior radiologist to find which predicted reports are described the given images more accurately.

### Training details

The whole network is implemented using a PyTorch framework based on Python 3.6 and trained on two GeForce RTX 2080Ti GPUs. We adopt DenseNet-121 with no pretraining as the backbone to extract visual features. There are three steps in our training process: external auxiliary signal-guide pretraining, DenseNet pretraining, and internal auxiliary guide training. In the first step, the maximum length of the sentence is 300 (padded with 0s), and the word embedding dimension is 300. We train ASGK for 30 epochs until convergence. The natural language decoder consists of three blocks. We adopt ADAM for optimizing and the training rate is 5e-4. For the second step, we resize the image to $224\times 224$ for both global and region images. The batch size is 32. We jointly train two DenseNets for 50 epochs until convergence. The learning rate starts from 1e-2 and delays by 0.1 every 10 epochs until 1e-5. We threshold the heat map by 0.7 to acquire region images. We adopt the model that achieves the best performance on test datasets as a visual extractor in the third step. In the final step, we resize the images to $224\times 224$ and train the entire network for 30 epochs until convergence. The learning rates for the visual extractor and ASGK are 1e-5 and 5e-4, respectively. We also adopt the ADAM optimizer to minimize the loss function. Among the multi-tasks, we set all loss weights to 1.

## Results and analysis

**Table 2:** Evaluation metrics on CH-CHR and COV-CTR datasets comparing ASGK with other methods

Dataset	Model	C	R	B@1	B@2	B@3	B@4	Hit(%)
CX-CHR	CoAtt	273.5	**64.5**	64.7	57.5	52.5	48.7	8.0
	HRGR	289.5	61.2	67.3	58.7	53.0	48.6	−
	KERP	285.0	61.8	67.3	58.8	53.2	47.3	−
	V-BERT	302.4	63.7	**68.6**	60.1	54.1	50.3	19.0
	V-GPT	301.8	63.0	67.9	59.6	54.0	48.7	−
	SAT	311.2	63.3	62.3	55.2	53.9	48.1	−
	R2Gen	310.2	63.3	68.1	60.2	54.3	50.1	−
	Ours	**324.5**	64.1	**68.6**	**60.8**	**55.8**	**52.3**	**20.0**
COV-CTR	CoAtt	67.2	74.8	70.9	64.5	60.3	55.2	25.0
	SAT	65.9	72.3	69.7	62.1	56.8	51.5	−
	AdaAtt	68.2	72.6	67.6	63.3	59.6	51.4	−
	V-BERT	**68.4**	74.7	71.0	65.3	60.6	55.8	26.0
	V-GPT	68.0	**74.6**	70.8	64.5	60.0	54.9	−
	R2Gen	67.2	73.2	69.3	61.1	55.9	51.8	−
	TopDown	63.1	72.1	70.5	65.3	60.9	56.1	−
	Ours	**68.4**	**74.6**	**71.2**	**65.9**	**61.1**	**57.0**	**27.0**

C and R are short for CIDER-D and ROUGE-L. B-n denotes that the BLEU score uses up to n-grams. Hit represents the human evaluation results

The bold numbers are the largest in each column

### Automatic evaluation

Table 2 summarizes the performances on the automatic evaluation metrics of different models. The results on both datasets indicate that ASGK outperforms all existing state-of-the-art models through its exploitation of auxiliary signals to guide the framework in knowledge pretraining and knowledge transfer procedures. The results demonstrate the robustness and superior generalization ability of ASGK. We also combine our medical graph encoder with V-Bert [5] and V-GPT [31] in order to validate the capability of the language-to-vision transfer. We adopt CIDER-D as the main metric to validate our model. On the large-scale CX-CHR dataset, ASGK significantly boosts performance compared with other baselines, it increases the CIDER score by 51.0, 35.0, 39.5, 22.1 and 22.7 respectively. However, ASGK only acheives a slightly low ROUGE-L score than the CoAtt [13] method. ASGK also outperforms other baselines in COV-CTR dataset.

Compared with the results present in Table 3, ASKG performed better than TieNet [38], CARG [25], SentSAT [43] and SentSAT+KG [45]. The most Cider score indicates that our generated reports have the least redundancy as there are many similar sentences used in each medical report to describe the normal terminology in which patients care less.

**Table 3:** Comparison of report generation models on three metrics on the Open-IU dataset

Model	Bleu-4	Cider-D	Rouge-L
CARG [25]	11.3	−	35.4
KERP [17]	**16.2**	28.0	33.9
TieNet [38]	8.1	−	31.1
SentSAT [43]	14.3	26.8	35.9
SentSAT+KG [45]	14.7	30.4	**36.7**
Ours	12.5	**30.6**	27.9

As some of their works are outsourced, we directly use the results reported in their papers

The bold numbers are the largest in each column

### Medical tags classification

The AUCs of medical tag classification, which contains both normal and abnormal terminologies on both datasets, are presented in Table 4. Our framework, which is guided by two auxiliary signals, outperforms the baseline on both datasets. Baseline outputs are predicted by a DenseNet-121 without pretraining. We attempt to boost the performance through the use of internal auxiliary signals and the adaptation of focal loss to balance the deviation. This demonstrates that internal auxiliary signals effectively promote the medical graph encoder and facilitate the medical tag classification.

### Human evaluation

Given 200 random images from these two datasets equally, we invited three radiologists to evaluate the corresponding outputs of our methods, CoAtt [13] and Vison-Bert [5]. They are encouraged to select a more accurate result from each pair. The human evaluation results are presented in Table 2. It shows that in the CX-CHR and COV-CTR datasets, radiologists thought $20\%$, and $27\%$ portions of our reports are more accurate than others’ respectively, and while they thought $53\%$, and $22\%$ portions of results are same. The human evaluation demonstrates that our method is capable of generating accurate and semantic-coherent reports.

**Fig. 4:**
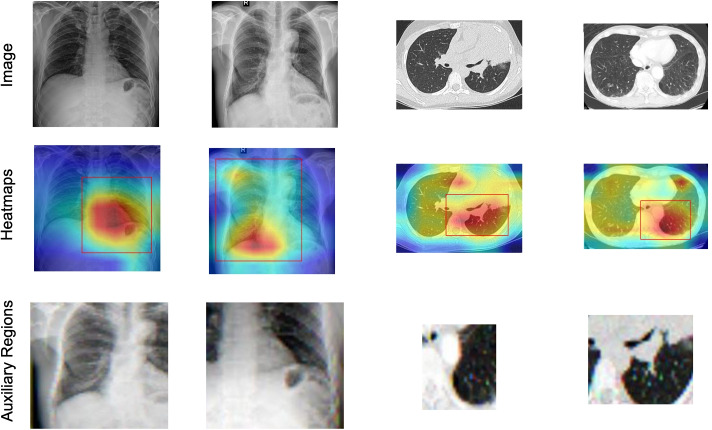
Sample output of our approach on both CX-CHR and COV-CTR datasets. We use the outputs before the last pooling layer in DenseNet-121 to generate heat maps, then threshold them by $\tau =0.7$ to produce the suspicious regions

**Fig. 5:**
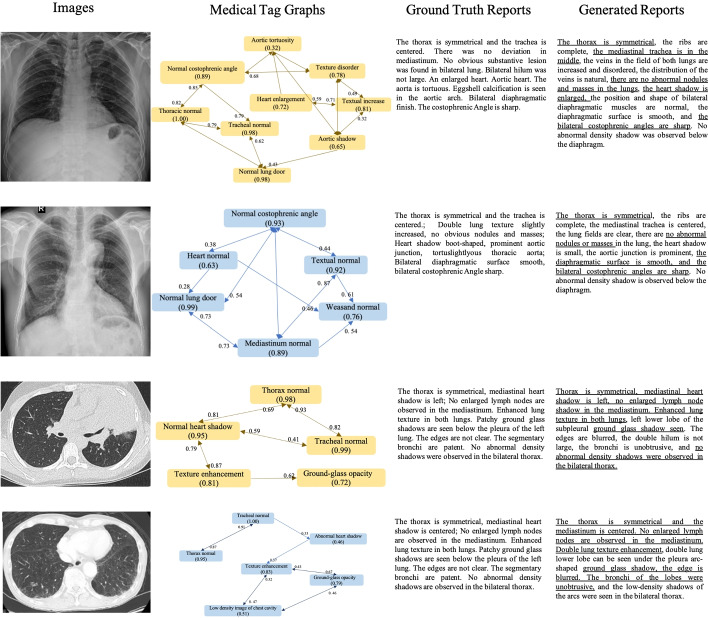
Sample output of our approach on both CX-CHR and COV-CTR datasets. In the medical tag graphs, we show the nodes whose value (which is equal to the classification probability) exceeds 0.5 and edges whose weights are more than 0.3. To read the image clearly, we show the values of some edges in the appropriate places. The underlined text indicates alignment between ground truth reports and generated reports

### Visualization

An illustration of heat maps, suspicious regions, is presented in Fig. 4. It is clear from the results that suspicious regions suggest the region on which the model should focus. For example, in the first row, the auxiliary region focuses on the inferior lobe of the left lung which presents a shadow. In the fourth row, moreover, the auxiliary region focuses the inferior pleural of the left lung, which covers ground-glass opacity, one of the symptoms of COVID-19.

Figure 5 shows the illustration of medical tag graphs, and paragraphs of medical reports. The medical tag graph demonstrates that ASGK is capable of encoding input features into a high-level knowledge graph; as we lack the ground truth of the corresponding graph, we train in an end-to-end way to encode the graph. The generated reports demonstrate the high quality and provide significant alignment with the ground truth.

### Ablation studies

We conduct ablation experiments to compare the performance of the two auxiliary signals. Table 4 presents the results of automatic evaluation metrics and tag classification. The baseline represents the direct training of the ASGK model without any auxiliary signals. In addition to extra notes, we adopt focal loss as our training strategy.

**Table 4:** Ablation studies for different auxiliary signals

Dataset	Model	CIDER-D	ROUGE-L	BLEU-4	AUC
CX-CHR	baseline	289.7	61.3	48.3	78.7
	baseline+IA+CE	304.6	62.5	48.9	82.1
	baseline+IA	305.3	62.7	49.1	83.2
	baseline+EA	317.2	63.8	52.0	79.3
	baseline+IA+EA	**324.5**	**64.1**	**52.3**	**85.9**
COV-CRT	baseline	59.1	68.3	52.5	72.7
	baseline+IA+CE	61.3	70.2	54.1	79.0
	baseline+IA	62.8	70.5	54.2	79.7
	baseline+EA	66.9	72.0	55.6	74.5
	baseline+IA+EA	**68.4**	**74.6**	**57.0**	**80.4**

IA, EA and CE are short for “internal auxiliary signals”, “external auxiliary signals’ and “cross entropy”. Four metrics are adopted to evaluate our model on two datasets

The bold numbers are the largest in each column

#### Do internal auxiliary signals help?

From Table 4, we can determine that auxiliary signals significantly boost the tag classification performance and improve the quality of generated reports. The internal auxiliary signal-guided learning outperforms the automatic metrics $15.6\%$, $1.4\%$ and $0.6\%$ respectively, and also performs $4.5\%$ better than the baseline in terms of classification accuracy on the CX-CHR dataset. The quality of the medical tag graphs significantly impacts the natural language decoder. We produce internal auxiliary signals to mimic radiologists’ working patterns, since abnormal regions provide richer visual features. These experiments demonstrate that focusing on abnormal regions benefits the detection of medical tags and the generation of medical reports.

#### What is the use of focal loss?

Radiologists are asked to describe all of their observations on one medical image, which leads to serious data deviation on medical tag labels and reports. Typically, each image contains three to five normal tags and a few abnormal terminologies. To alleviate the deviation in multi-label classification tasks, we adopt focal loss in order to optimize the parameters in DenseNet and the medical tag decoder. When the second and third rows are compared, the performance shows its capability to balance deviation and improve AUC metrics. Without focal loss, the performances on AUC metrics decrease by $0.9\%$ and $0.7\%$ respectively on the two datasets.

#### Are external auxiliary signals useful?

The external auxiliary signals guide the pretraining procedure to assist the model in memorizing and phrasing medical knowledge. As expected, ASGK benefits a lot from the pretraining procedure. The performance on automatic metrics are boosted substantially from $289.7\%$ to $317.2\%$ and $59.1\%$ to $66.9\%$ on the two datasets respectively, which indicates that external auxiliary signal-guided training is capable of generating accurate and semantically coherent sentence. However, it improves the classification accuracy slightly, by $0.6\%$, and $1.8\%$ respectively on the two datasets, which demonstrates that exploiting medical domain knowledge primarily promotes the natural language decoder. Furthermore, our findings show that without external auxiliary signals, the model fails to alleviate the data bias and is therefore prone to repeating several specific words and sentences in one report.

Overall, the internal signals mainly facilitate the medical tag encoder’s effectiveness in generating fine-grained sentences and describing more medical tags. The external signals enable the natural language decoder to generate more semantically coherent sentences.

## Broader impacts

This work practically analyzes a meaningful task combined with the computer vision and natural language processing task, medical report generation. Especially when pandemic happens like COVID-19, robust and accurate medical report generation technology is of great clinical value, which can reduce the burden on doctors and enable people to more accurately grasp their health status. We propose an anthropomorphic model, mimicking radiologists’ working patterns, to promote the medical report generation task via acquiring easily-accessed auxiliary signals. This approach may inspire those researchers who have limited access to medical image resources to dig deeper into adopting unsupervised learning methods to acquire more auxiliary signals to supervised this task and achieve state-of-the-art performances. However, it still needs more effort to provide theoretical interpretation for these auxiliary signals. And our algorithm should be utilized carefully in clinical practice since medical decisions may lead to live-or-death consequences.

## Conclusions and future work

In this paper, we proposed an Auxiliary Signal-Guided Knowledge Encoder-Decoder approach that mimics radiologists’ working patterns to generate fine-grained and semantically coherent medical reports. We investigated how to best crop the auxiliary region from the global medical image, how to exploit medical domain knowledge from medical textbook, and how these auxiliary signals work. Experiments demonstrate that ASGK outperforms existing methods and boosts the performance of medical report generation tasks on report generation and tag classification on two medical datasets. Moreover, we have constructed and released a new medical report dataset, COV-CTR, to contribute to the community. In the future, we plan to focus on building a general captioning framework guided by auxiliary signals to encode and decode general corpora knowledge.
